# Plasma Protein Binding, Biostability, Metabolite Profiling, and CYP450 Phenotype of TPB15 Across Different Species: A Novel Smoothened Inhibitor for TNBC Therapy

**DOI:** 10.3390/pharmaceutics17040423

**Published:** 2025-03-26

**Authors:** Dingsheng Wen, Boyu Chen, Mingtong Deng, Shaoyu Wu, Shuilin Xie

**Affiliations:** 1 Key Specialty of Clinical Pharmacy, The First Affiliated Hospital of Guangdong Pharmaceutical University, Guangzhou 510080, China; wen_dingsheng@gdpu.edu.cn; 2 School of Pharmacy & Clinical Pharmacy (Integrated School of Pharmacy), Guangdong Pharmaceutical University, Guangzhou 510006, China; 2201901109@gdpu.edu.cn; 3 School of Pharmaceutical Sciences, Southern Medical University, Guangzhou 510515, China; cby931939709@smu.edu.cn; 4 School of Biological Science and Engineering, South China University of Technology, Guangzhou 511436, China

**Keywords:** TPB15, metabolic enzyme phenotype, plasma protein binding, species different, metabolite

## Abstract

**Background/Objectives**: Triple-negative breast cancer (TNBC) is a major cause of cancer-related deaths among women. The Hedgehog (Hh) signaling pathway plays a critical role in tumor development, and targeting this pathway may provide new therapeutic opportunities for TNBC. TPB15 is a novel smoothened inhibitor of the Hh pathway, showing promising tumor reduction and low-toxicity properties in vivo/vitro. This study aims to evaluate TPB15’s protein binding rates, metabolic stability, and metabolism across different species, including mice, rats, dogs, monkeys, and humans. **Methods**: TPB15 was synthesized, and its pharmacokinetic profile was assessed. Plasma protein binding was determined using ultrafiltration across multiple species. Stability studies were conducted in plasma and liver microsomes from each species. Additionally, metabolic enzymes in human liver microsomes were characterized with selective CYP450 inhibitors, and high-resolution mass spectrometry was employed to identify metabolites. **Results**: Plasma protein binding of TPB15 was consistent across species, ranging from 81.5% to 82.4% in humans and rats. After 120 min, TPB15 remained stable in plasma, with retention rates of 97.2–98.3%. The elimination half-life (*t*_1/2_) varied from 88 min in monkeys to 630 min in dogs. In human liver microsomes, metabolism was significantly inhibited by sulfaphenazole and ketoconazole, indicating the involvement of CYP3A4 and CYP2C9 enzymes. TPB15 underwent phase I metabolism, producing a major metabolite with a molecular weight of 468.9. **Conclusions**: TPB15 demonstrates stable pharmacokinetic properties across species, with consistent protein binding and significant variability in half-life. The observed differences in metabolism are primarily attributed to CYP2C9 and CYP3A4, offering valuable insights into its drug development potential.

## 1. Introduction

Tumors represent a significant threat to human health and are the second leading cause of death worldwide [1]. Among these, triple-negative breast cancer (TNBC) is one of the most prevalent malignant tumors and the primary cause of cancer-related mortality among women. Treatment options for patients include surgery, radiotherapy, chemotherapy, and targeted drug therapy [2]. Despite advancements in radiation and chemotherapy, the challenges of drug resistance and tumor recurrence continue to hinder effective treatment, rendering TNBC a particularly formidable disease [3]. Given its severe impact on women’s health, the development of novel therapeutics for TNBC is an urgent priority.

The Hedgehog (Hh) signaling pathway is a crucial signaling cascade that transmits information from the cell membrane to the nucleus, playing a vital role in normal embryonic development [4,5]. This pathway functions through a series of signaling events that ultimately alter the balance between the activator and repressor forms of the Glioma-associated oncogene (Gli) transcription factors. Abnormal activation of the Hh signaling pathway can occur due to mutations in related genes (ligand-independent signaling) or the overexpression of Hh signaling molecules (ligand-dependent signaling—either autocrine or paracrine), involving three main types of proteins: Hh ligands, Ptched protein (Ptch), and Smoothened (Smo) receptors [6,7]. The Smo receptor, a seven-transmembrane protein structurally akin to G protein-coupled receptors (GPCRs), serves as a key regulator of the Hh signaling pathway [8,9,10,11]. Dysregulated Hh signaling is implicated in the growth, proliferation, and invasion of tumor cells, contributing significantly to tumorigenesis. Consequently, the development of efficient and low-toxicity Smo-targeted inhibitors has emerged as a primary focus in the search for effective TNBC therapies, holding considerable promise for clinical application.

**Figure 1:**
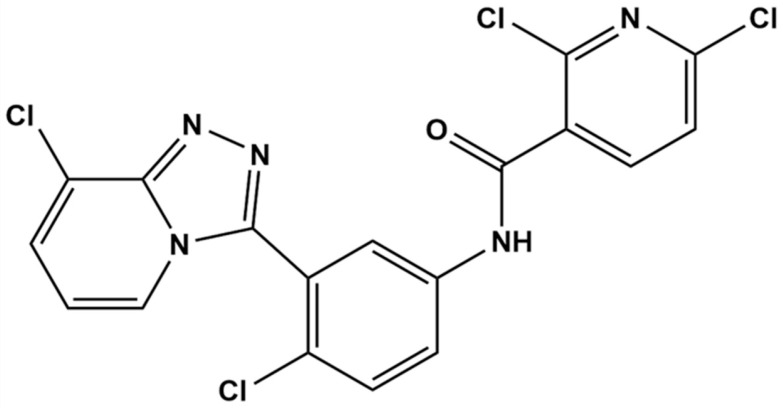
Structural formula of compound TPB15.

TPB15 is a pyridine–triazole compound developed by our research team, with its structural formula depicted in Figure 1. Preliminary studies indicate that TPB15 effectively binds to the Smo target and inhibits Hh signaling activation. Both in vitro and in vivo experiments have demonstrated its potent anti-TNBC effects. In vitro studies reveal that TPB15 significantly inhibits the proliferation of TNBC cell lines (MDA-MB-468, MDA-MB-231), suppresses clonal formation, and induces senescence and apoptosis in breast cancer cells. The half-maximal inhibitory concentration (*IC*_50_) of TPB15 on normal mammary epithelial cells (MCF10A) is 169 µM, suggesting low toxicity to normal cells. In vivo pharmacodynamic studies using the MDA-MB-468 xenograft nude mouse model indicate that TPB15 exhibits a robust tumor inhibitory effect, surpassing that of the positive control drug Vismodegib. Furthermore, acute toxicity studies reveal a lethal dose for 50% of the population (LD_50_) of TPB15 in mice at 3875.35 mg/kg, indicating relatively low toxicity in vivo according to the Globally Harmonized System of Classification and Labelling of Chemicals (GHS) [12]. Pharmacokinetic studies in rats have demonstrated that TPB15 possesses a relatively long half-life, indicating good persistence and stability. Given the results of TPB15’s in vitro and in vivo pharmacodynamic experiments and safety assessment, TPB15 has the potential to be developed into a novel anti-triple-negative breast cancer drug. Despite these promising findings, there remains a lack of comprehensive understanding regarding the metabolic products of TPB15 and the potential pharmacokinetic differences across species [13].

This study aims to conduct preclinical pharmacokinetic research on TPB15, elucidating its pharmacokinetic characteristics, including plasma protein binding rate, metabolic stability, metabolic phenotype, and identification of metabolites. The outcomes of these studies provide substantial evidence supporting TPB15’s favorable drug metabolism and pharmacokinetic properties as a candidate for clinical development.

## 2. Materials and Methods

### 2.1. Chemical and Reagents

TPB15 was synthesized at the School of Pharmacy, Southern Medical University (Guangzhou, China) [12]. Tinidazole was purchased from Guangzhou Jiajia Tu Biotechnology Co., Ltd. (Guangzhou, China). Plasma and liver microsomes from five species—human, monkey, dog, rat, and mouse—were provided by Jiangsu Kejing Bio-Pharmaceutical Co., Ltd. (Changzhou, China). Formic acid, methanol, acetonitrile, and ammonium acetate were obtained from Merck (Darmstadt, Germany). Ethyl acetate, methyl tert-butyl ether, and thiabendazole hydrochloride were purchased from Sigma-Aldrich (St. Louis, MO, USA). PBS buffer solution (dry powder) was sourced from Wuhan Saiwei Biotechnology Co., Ltd. (Wuhan, China), while dimethyl sulfoxide was acquired from Beijing Jinclone Bio-Technology Co., Ltd. (Beijing, China). Coumarin, quinidine, quercetin, α-naphthoflavone, sulfamethoxazole, and ketoconazole were all obtained from MedChemExpress (Monmouth Junction, NJ, USA). NADPH and MgCl₂ used in the experiments were purchased from Beijing Solaibao Technology Co., Ltd. (Beijing, China) and Shanghai Puzhen Bio-Technology Co., Ltd. (Shanghai, China), respectively.

### 2.2. Methods

#### 2.2.1. The Plasma Protein Binding Rates of TPB15 in Plasma from Mice, Rat, Dog, Monkey, and Human

To prepare plasma samples of TPB15 at final concentrations of 4, 8, and 16 μg/mL, 1 μL of TPB15 dissolved in dimethyl sulfoxide (DMSO) at low (400 μg/mL), medium (800 μg/mL), and high (1600 μg/mL) concentrations was added to 99 μL of blank plasma obtained from humans, rats, mice, monkeys, and dogs. Each concentration was prepared in six replicates. The samples were then divided into two groups: one for determining the pre-ultrafiltration concentration (Cpre) and the other for measuring the post-ultrafiltration concentration (Ct), following the evaluation of the non-specific binding coefficient (NSB) of TPB15 to the ultrafiltration tubes. For Cpre determination, 10 μL of the sample was mixed with 990 μL of plasma, vortexed thoroughly, and analyzed for drug concentration using the previously reported liquid chromatography–tandem mass spectrometry (LC-MS/MS) method [13]. For Ct determination, the samples were first incubated in a water bath at 37 °C for 3 h. After incubation, they were transferred to ultrafiltration tubes and centrifuged at 14,000 rpm for 20 min at 4 °C using a centrifuge (Sigma 3K-15) from Sigma Laborzentrifugen GmbH (Osterode am Harz, Germany). After centrifugation, 10 μL of the ultrafiltrate was mixed with 990 μL of plasma, vortexed, and analyzed for concentration using the same LC-MS/MS method. The formula for calculating the plasma protein binding rate (PPB) is as follows: PPB (%) = [1 − Ct/(1 − NSB)/Cpre] × 100%. (NSB: non-specific binding coefficient. Cpre: post-ultrafiltration concentration. Ct: pre-ultrafiltration concentration).

#### 2.2.2. Stability of TPB15 in Plasma from Mice, Rat, Dog, Monkey, and Human

A total of 696.5 μL of plasma from each species (healthy mice, rat, dog, monkey, and human) were spiked with 3.5 μL of TPB15 stock solution (45.3 mg/L in DMSO), resulting in a final TPB15 concentration of 453 μg/L in plasma. The mixture was then aliquoted into three tubes, each with a volume of 200 μL, maintaining a final DMSO concentration of 0.5%. After incubation in a water bath at 37 °C for 0, 30, 60, 90, and 120 min, 100 µL samples were transferred into EP tubes. Subsequently, 10 µL of internal standard working solution were added, followed by vortex mixing. Then, 500 µL of ethyl acetate were added, and the mixture was vortexed again before being centrifuged at 14,800 rpm at 4 °C for 10 min. A total of 400 µL of the supernatant were transferred into a new EP tube and placed in a vacuum concentrator (Bionoon-VAC2) (Bionoon Biotech, Shanghai, China) to dry for 1 h. The dried residue was reconstituted with 100 µL of mobile phase, vortex mixed thoroughly, and centrifuged again at 14,800 rpm at 4 °C for 10 min. A volume of 5 µL of the supernatant was then taken for LC-MS/MS analysis to determine the drug concentration. Additionally, a blank control group was established, in which PBS buffer was used in place of blank plasma in the incubation system.

#### 2.2.3. Metabolic Stability of TPB15 in Liver Microsome from Mice, Rat, Dog, Monkey, and Human

A total of 450 μL liver microsomes from mice, rat, dog, monkey, and human were spiked with 0.5 μL of TPB15 solution and pre-incubated in a water bath at 37 °C for 10 min. Following this, 50 μL of NADPH were added to initiate the reaction. The concentrations of each substance in the incubation system are detailed in Table 1. After initiating the metabolic reaction, 50 μL aliquots were removed at incubation times of 0, 5, 15, 30, 45, 60, and 90 min and transferred to 150 μL of icy methanol solution containing the internal standard to terminate the reaction. The mixtures were swirled for 2 min and then centrifuged at 14,800 rpm at 4 °C for 10 min. A volume of 130 μL of the supernatant was transferred into a new EP tube and centrifuged again at 14,800 rpm at 4 °C for 10 min, and 5 μL of the supernatant were injected for the determination of substrate TPB15 concentration. Three samples were prepared in parallel at each time point. Additionally, both a positive control group and a blank control group were established. In the positive control group, substrate testosterone was incubated with liver microsomes from mice, rat, dog, monkey, and human. The concentrations of substrate testosterone were measured at 0 and 90 min, and the changes in concentration before and after incubation were compared to confirm the activity of the liver microsomes. The blank control group included one drug-containing inactivated liver microsome sample for each species. The metabolic stability parameters, including in vitro elimination half-life (*T*_1/2_) and in vitro intrinsic clearance rate (*CL*_int_), were calculated based on this graph using the following formulas: in vitro elimination half-life (*T*_1/2_) = −0.693/k and in vitro intrinsic clearance rate (*CL*_int_) = (0.693 × incubation medium (mL))/(liver microsomes (mg) × T_1/2_).

#### 2.2.4. Metabolic Enzyme Phenotypes in Human Liver Microsomes

The specific inhibitors of P450 were employed to evaluate the metabolic phenotype of TPB15, and the metabolic enzymes corresponding to each inhibitor and the system concentration are shown in Table 2. The specific procedure was as follows: a total of 0.5 μL of the substrate TPB15 and 0.5 μL of each specific inhibitor were mixed with 449 μL of human liver microsomes, and the mixture was incubated at 37 °C for 10 min. Subsequently, 50 μL of NADPH with a concentration of 10 mM in PBS were introduced to initiate the reaction. After 90 min, 50 μL of the reaction mixture were transferred to a 150 μL ice-cold methanol solution containing an internal standard to terminate the reaction. The mixture was vortexed for 2 min and then centrifuged at 4 °C at 14,800 rpm for 10 min. A volume of 130 μL of the supernatant was then transferred to a new EP tube, which was centrifuged again at 4 °C at 14,800 rpm for another 10 min. Finally, 5 μL of the supernatant were injected for the determination of substrate TPB15 concentration. Three parallel samples were prepared for each time point. The experiment also included a positive control group and a negative control group. In the positive control group, substrate TPB15 was incubated with human liver microsomes for 90 min without the addition of inhibitors. In the negative control group, the substrate TPB15 was incubated for 90 min without inhibitors, with human liver microsomes replaced by an equal volume of phosphate-buffered saline (PBS). The metabolic rate of TPB15 in human liver microsomes was represented by the elimination rate of the substrate TPB15. Data processing and plotting were conducted using GraphPad Prism 9.0 and SPSS 20.0 software. The inhibition rate was calculated using the following formula: inhibition rate (%) = [1 − (concentration of the negative control group − concentration of the experimental group)/(concentration of the negative control group − concentration of the positive control group)] × 100%.

#### 2.2.5. Metabolite Identification

A total of 450 μL of human liver microsomes were spiked with 0.5 μL of the substrate TPB15 and incubated in a water bath at 37 °C for 10 min. The reaction was initiated by adding 50 μL of NADPH. Following incubation, 50 μL of the reaction mixture were transferred to 150 μL of ice-cold methanol to terminate the reaction. The mixture was then centrifuged at 14,800 rpm at 4 °C for 10 min. A volume of 5 μL of the supernatant was collected for analysis using a QTRAP 4000 mass spectrometer(AB Sciex, Framingham, MA, USA), with three parallel samples prepared for each time point. The primary mass spectrometry conditions were as follows: curtain gas at 30 psi, sprayer gas (GS1) at 50 psi, drier gas (GS2) at 50 psi, ion source temperature at 550 °C, and cluster-removal voltage at 100 V. The ion source (IS) spray voltage was set to 5500 V, with a focusing potential (FP) of 200 V, an entrance potential (EP) of 10 V, an exit potential (CXP) of 15 V, and a collision energy of 86 V for TPB15. Chromatographic separation was performed using an Agilent ZORBAX Stable Bond C18 column (50 mm × 2.1 mm i.d., 3.5 μm particle size) at a column temperature of 35 °C. The injection volume was 5 μL, and the elution was carried out in gradient mode. The mobile phase consisted of two components: mobile phase A (an aqueous solution with 0.1% formic acid) and mobile phase B (methanol). The elution gradient was as follows: 0.3 min at 10% B, 3.0 min at 95% B, 4.5 min at 95% B, and 4.6 min at 10% B, yielding a total analysis time of 7 min.

#### 2.2.6. Statistical Analysis

The Drug and Statistics 2.0 (DAS 2.0) software package (Mathematical Pharmacology Professional Committee of China, China) was used to calculate the pharmacokinetic parameters for TPB15. All data are shown as mean ± standard deviation (s.d.). Statistical significance was conducted by one-way analysis of variance (ANOVA), with a value of lower than 0.05.

## 3. Results

### 3.1. The Plasma Protein Binding Rates of TPB15 in Plasma from Mice, Rat, Dog, Monkey, and Human

#### 3.1.1. The Non-Specific Adsorption Coefficient of TPB15 to the Ultrafiltration Tubes

Table 3 summarizes the non-specific binding (NSB) coefficients of TPB15 with ultrafiltration tubes, measured using plasma ultrafiltrate at low, medium, and high concentrations. The NSB values obtained were (1.75 ± 0.16)%, (2.76 ± 0.27)%, and (2.67 ± 0.34)%, respectively, leading to an average NSB of (2.39 ± 0.46)%. These results indicate that TPB15 has low adsorption to the ultrafiltration tube membrane.

#### 3.1.2. The Plasma Protein Binding Rates

The plasma protein binding rates of TPB15 across different species were calculated using the previously mentioned formula, with results detailed in Table 4. At low, medium, and high concentrations, the plasma protein binding rates of TPB15 consistently exceeded 80%. Specifically, the average binding rates ranged from 81.51% to 82.15% in human plasma, from 80.87% to 82.40% in rat plasma, from 81.21% to 82.40% in mouse plasma, from 81.24% to 82.20% in monkey plasma, and from 81.03% to 82.28% in dog plasma. Overall, the plasma protein binding rate was approximately 80.87% to 82.40%. These findings suggest that TPB15 exhibits high protein binding among all species examined, with no concentration dependence observed. Furthermore, significant differences in the plasma protein binding rates among the five species were not detected, and binding rates within the concentration range of 4 to 16 μg/mL remained relatively stable.

### 3.2. Stability of TPB15 in Plasma from Mice, Rat, Dog, Monkey, and Human

Table S1 in Supplementary summarizes the stability results for TPB15 in plasma. After a 120 min incubation in plasma from various species, the remaining concentrations of TPB15 were (97.2 ± 1.1)% in human plasma, (98.0 ± 0.6)% in rat plasma, (98.0 ± 1.2)% in mice plasma, (98.3 ± 1.5)% in monkey plasma, and (98.0 ± 0.8)% in dog plasma. These results demonstrate that TPB15 is stable in plasma, with no significant differences observed among the five species tested.

### 3.3. Metabolic Stability of TPB15 in Liver Microsome from Mice, Rat, Dog, Monkey, and Human

#### 3.3.1. Positive Drug Control Groups in Liver Microsomes from Various Species

**Figure 2:**
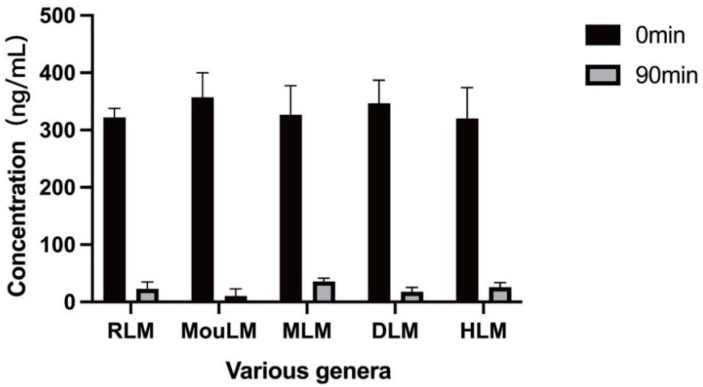
Metabolic residual concentration maps of testosterone in various types of liver microsomes (Sprague-Dawley rat microsomes (RLM), KM mice microsomes (MouLM), beagle dog microsomes (DLM), crab-eating monkey microsomes (MLM), and human microsomes (HLM)).

The results indicate that after 90 min of incubation, testosterone was metabolized in the liver microsomes of Sprague-Dawley rats (RLM), KM mice (MouLM), beagle dogs (DLM), crab-eating macaques (MLM), and humans (HLM). These findings confirm the normal activity of liver microsomes across the five species, facilitating subsequent metabolic studies of TPB15. The metabolic data for testosterone in liver microsomes from various species are shown in Table S2 in Supplementary, with the remaining concentration graph represented in Figure 2.

#### 3.3.2. Metabolic Stability of TPB15 in Liver Microsome from Mice, Rat, Dog, Monkey, and Human

**Figure 3:**
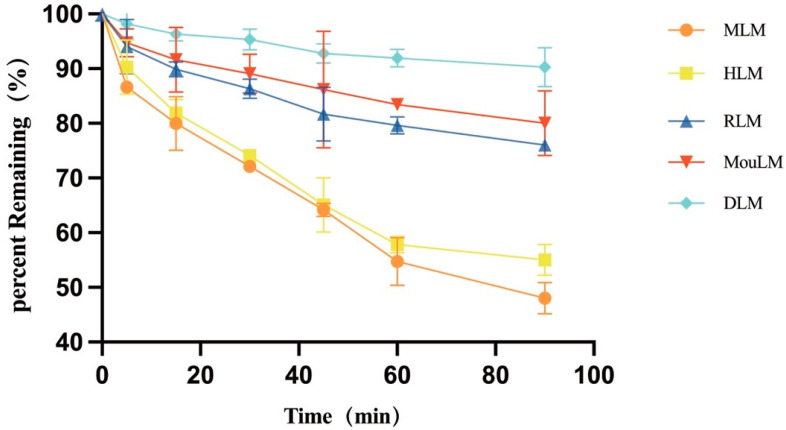
Metabolic stability profile of TPB15 in various liver microsomes (RLM: Sprague-Dawley rat microsomes, MouLM: KM mice microsomes, DLM: beagle dog microsome, MLM: crab-eating monkey microsomes, HLM: human microsomes).

**Figure 4:**
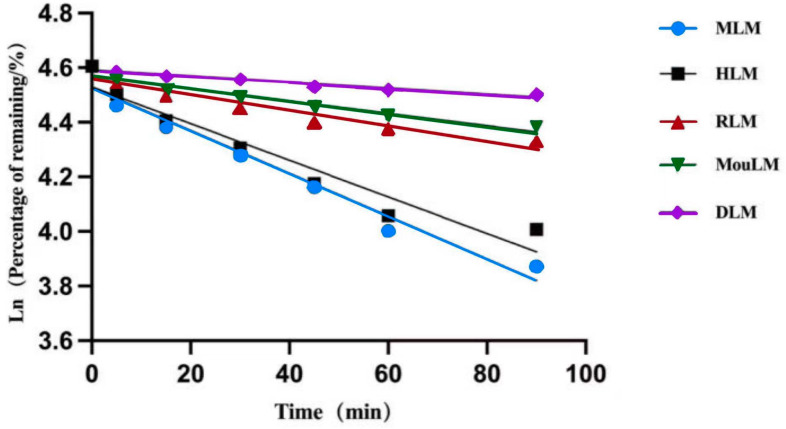
Linear regression plots (RLM: Sprague-Dawley rat microsomes, MouLM: KM mice microsomes, DLM: beagle dog microsome, MLM: crab-eating monkey microsomes, HLM: human microsomes).

After a 90 min incubation, TPB15 was metabolized in liver microsomes from Sprague-Dawley rats, KM mice, beagle dogs, crab-eating monkeys, and humans. The metabolic stability curve of TPB15 was generated by plotting substrate concentration against time, as illustrated in Figure 3. The data indicate that the remaining proportions of TPB15 were 90.3 ± 3.54% in beagle dog microsomes, 80.0 ± 5.93% in KM mice, 76.0 ± 0.89% in Sprague-Dawley rats, 55.0 ± 2.81% in humans, and 48.0 ± 2.86% in crab-eating monkeys after 90 min. These results suggest that TPB15 is metabolized more extensively in the liver microsomes of crab-eating monkeys and humans, and to a lesser extent in those of Sprague-Dawley rats, KM mice, and beagle dogs. The concentration of TPB15 at 0 min was considered 100%, with subsequent time points representing the remaining percentage. The natural logarithm of the remaining concentration was regressed linearly against incubational time, as shown in Figure 4, indicating a favorable linear elimination of TPB15 in liver microsomes from various species. Metabolic stability parameters were calculated based on this graph, utilizing the following formulas: in vitro elimination half-life (T_1/2_) = −0.693/k and in vitro intrinsic clearance rate (CL_int_) = 0.693/T_1/2_ × incubation medium (mL)/liver microsomes (mg). The results for T_1/2_ and CL_int_ across species are presented in Table 5, showing values of 305.15, 241.55, 88.34, 630.00, and 103.25 min for beagle dogs, KM mice, Sprague-Dawley rats, humans, and crab-eating monkeys, respectively. The corresponding intrinsic clearance rates were 0.0344, 0.0273, 0.0941, 0.0132, and 0.0805 mL/min/mg. Notably, significant interspecies metabolic differences were observed, with humans and crab-eating monkeys exhibiting the most similar metabolic profiles.

### 3.4. Metabolic Enzyme Phenotypes in Human Liver Microsomes

**Figure 5:**
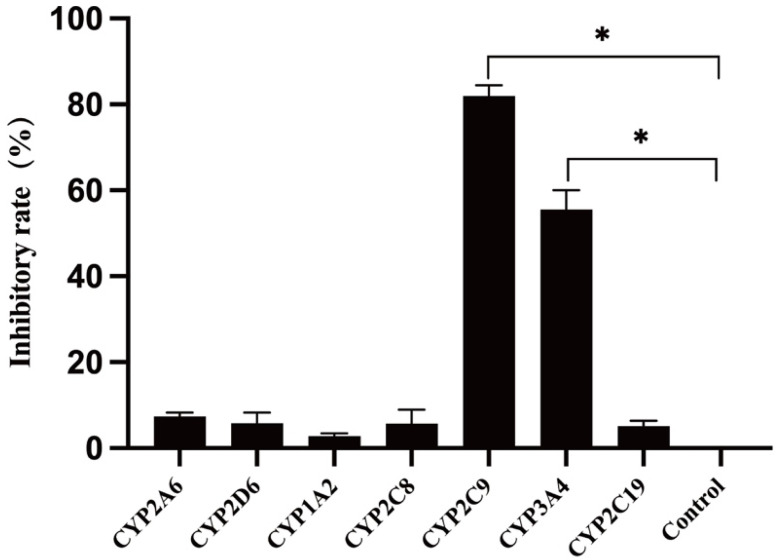
Effects of various inhibitors on TPB15 metabolism in human liver microsomes. Compared with control group, * *p* < 0.05.

The effects of various selective CYP450 enzyme inhibitors on TPB15 metabolism in human liver microsomes are detailed in Figure 5 and Table 6. Compared to the positive control group, the inhibitors sulfamethoxazole and ketoconazole significantly inhibited TPB15 metabolism (*p* < 0.05), with inhibition rates of 81.22% and 50.25%, respectively. Other inhibitors did not significantly impact TPB15 metabolic enzyme activity (*p* > 0.05), indicating that CYP2C9 and CYP3A4 are the primary metabolic enzymes involved in TPB15 metabolism in human liver microsomes.

### 3.5. Metabolite Identification

**Figure 6:**
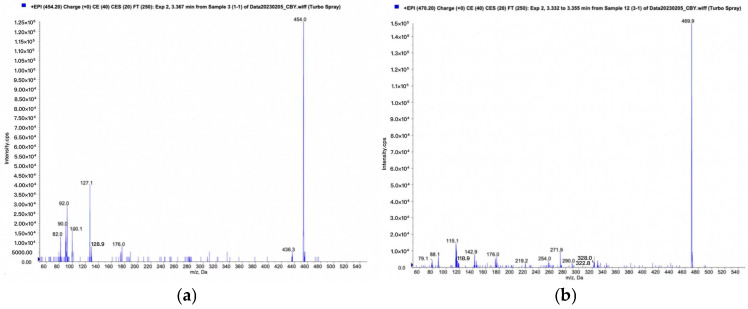
Human liver microsome incubated for 90 min: (**a**) TPB15 mass spectrum; (**b**) mass spectrum of metabolites.

**Figure 7:**
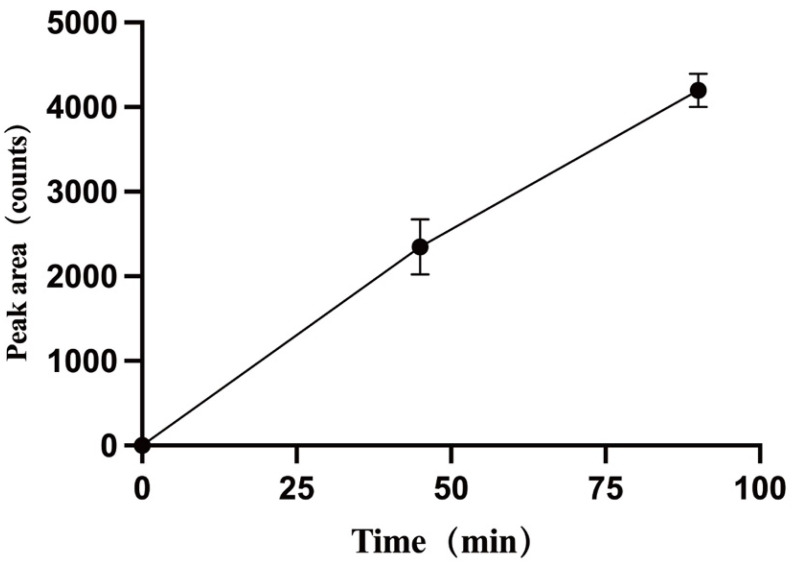
Plot of peak areas of metabolites against time.

Following the QTRAP workflow for metabolic product identification, screening modes of pMRM-IDA-EPI and Pre-IDA-EPI were established. Quantification of metabolites was performed using MultiQuant 3.0.3 software, with mass spectrometry diagrams for TPB15 and its metabolites depicted in Figure 6a,b. The precursor ion of TPB15 [M + H]+ exhibited an *m/z* of 453.9 and produced daughter ions at *m/z* of 127 and 100. A metabolite [M + H]+ was observed at an *m/z* of 469.9, yielding daughter ions with *m/z* of 143 and 116. This metabolite, not detected at 0 min, was observed in both the 45 min and the 90 min samples, with increasing amounts over time. Consequently, this product is identified as a metabolite of TPB15 in human liver microsomes. The peak area changes over time for this metabolite are illustrated in Figure 7. Compared to TPB15, the metabolite’s molecular weight increased by 16 Da, suggesting that the metabolic reaction of TPB15 in human liver microsomes involves oxidation, characterizing the metabolite as an oxidation product. Considering the structural features of TBP15, which contains a pyridine ring and a five-membered nitrogen-containing heterocycle, as well as the involvement of CYP3A4 and CYP2C9 in metabolism, we propose that oxidation most likely occurs at the N position of the pyridine ring. This hypothesis is supported by the metabolic characteristics of CYP enzymes: CYP3A4 and CYP2C9 preferentially catalyze oxidation at electron-rich sites, including aromatic and heteroaromatic rings. Pyridine oxidation typically occurs at the nitrogen atom (forming N-oxide) or at the C4 position due to its electronic properties. The observed time-dependent increase in the metabolite’s peak area further supports enzymatic oxidation rather than spontaneous degradation. While these findings strongly suggest oxidation at the pyridine C4 position, further structural characterization such as NMR analysis would be required for definitive confirmation.

## 4. Discussion

TPB15 is a new smoothened inhibitor to treat triple-negative breast cancer, showing remarkable activity in reduction in tumor volume, low toxicity in vivo/vitro, and good pharmacokinetic properties in rats [12,13]. However, animal models are commonly used in the preclinical development of new drugs to predict the metabolic behavior of new compounds in humans. It is, however, important to realize that humans differ from animals with regards to isoform composition, expression, and catalytic activities of drug-metabolizing enzymes [14]. Therefore, different species should be evaluated to find an animal model that is similar to humans for specific drugs in terms of metabolism stability and protein binding rate. In this study, the species differences in terms of protein binding rate and metabolism stability in plasma and liver microsomes in mice, rats, dogs, monkeys, and humans for TPB15 were determined, and human liver microsomes were employed to analyze the metabolic phenotype and metabolite identification, providing a reference for the safe use of drugs in clinic, the administration route, and the dosage of the drug, and laying a theoretical foundation for subsequent clinical pharmacokinetic studies.

Plasma protein binding (PPB) is a critical parameter that needs to be measured accurately, because it has significant impacts on the predictions of drug–drug interactions (DDI), estimations of therapeutic indices (TIs), and developments of PK/PD relationships [15]. In this study, the ultrafiltration method was employed to assess the PPB of TPB15 in the plasma of mice, rats, dogs, monkeys, and humans. TPB15 exhibited consistently medium plasma protein binding rates across all species tested, ranging from 80.87% to 82.40%. These findings are consistent with previous literature, which suggests that medium protein binding can influence the distribution and elimination of drugs within the body. The absence of significant concentration dependence in binding rates indicates that TPB15 maintains a stable pharmacokinetic profile, which is advantageous for therapeutic applications. Additionally, the lack of notable interspecies differences suggests that TPB15 may exhibit similar pharmacokinetic behavior across various animal models, making it a potentially suitable candidate for translational studies. It should be noted that while protein binding is important, the clinical manifestation of drug–drug interactions is often not observed in the absence of subsequent metabolism. This is because the unbound compound, once released from plasma proteins, may undergo metabolism by the cytochrome P450 (CYP450) enzyme system or be redistributed, leading to a reduction in its circulating concentration. Stability assessments revealed that TPB15 remained largely unchanged in plasma over a 120 min incubation period, with remaining concentrations exceeding 97% across all species. This stability is critical for ensuring that the drug maintains its therapeutic efficacy during circulation. The results corroborate findings from similar studies, where high stability in plasma is associated with reduced metabolism and degradation, thereby prolonging the drug’s half-life and enhancing its therapeutic window.

The metabolic stability of TPB15 varied among species, with significant differences observed in the remaining concentrations after a 90 min incubation in liver microsomes. Notably, TPB15 was metabolized more extensively in crab-eating monkeys and humans compared to other species. These findings are consistent with the notion that metabolic pathways can differ significantly between species due to variations in enzyme expression and activity [14]. The calculated elimination half-lives and intrinsic clearance rates further highlight these interspecies differences, with humans displaying a relatively lower intrinsic clearance rate, suggesting a slower metabolism of TPB15 compared to other species, which could explain why the half-life of TPB15 in rats was long [13]. The identification of CYP2C9 and CYP3A4 as the primary metabolic enzymes involved in TPB15 metabolism in human liver microsomes underscores the importance of cytochrome P450 enzymes in drug metabolism. The significant inhibition observed with sulfamethoxazole and ketoconazole confirms the involvement of these enzymes, which are known to play critical roles in the metabolism of various therapeutic agents [16]. This knowledge is essential for predicting potential drug–drug interactions and optimizing dosing regimens in clinical settings. The identification of TPB15 metabolites through high-resolution mass spectrometry adds another layer of understanding regarding its metabolic pathways. The observed oxidation of TPB15, indicated by the increase in molecular weight of the metabolite, suggests that oxidative metabolism is a key pathway for TPB15 in human liver microsomes. This finding is consistent with the metabolic behavior of many drugs that undergo phase I reactions [17]. The detection of metabolites over time highlights the dynamic nature of drug metabolism and the importance of continuous monitoring in pharmacokinetic studies.

This study provides valuable insights into TPB15’s pharmacokinetic properties, but certain limitations exist. The in vitro analyses using liver microsomes may not fully reflect in vivo conditions, and the functional activity and toxicity of the identified metabolite remain uncharacterized. Additionally, the phase II conjugation pathways were not examined. Despite these limitations, the study highlights TPB15’s potential as a clinical candidate for TNBC therapy, offering important information for animal model selection and predicting drug-drug interactions via CYP2C9/3A4. Future studies should prioritize in vivo validation in primates, structural elucidation of the metabolite, and investigation of phase II metabolism to facilitate clinical translation.

## 5. Conclusions

In summary, the findings from this study offer valuable insights into the pharmacokinetic properties of TPB15. The results demonstrate its stability in plasma and a moderate plasma protein binding rate, with no significant species differences observed among mice, rats, dogs, monkeys, and humans. The half-life of TPB15 in monkey microsomes was shorter than that in mice, rats, and dogs, but comparable to that in humans. This pharmacokinetic behavior is attributed to oxidation by CYP2C9 and CYP3A4, leading to the formation of a primary metabolite with a molecular weight of 468.9.

## Figures and Tables

**Table 1:** Concentration of each substance in the liver microsome incubation system.

	Substance	Systemic Concentration
Incubation system	Liver microsomes	0.5 mg/mL
	Drug (TPB15)	5 μM
	NADPH	1 mM
	MgCl_2_	3 mM
	PBS	100 mM

**Table 2:** Respective inhibitor corresponding metabolic enzymes versus inhibitor concentrations.

Inhibitor	Corresponding CYP Metabolic Enzymes	System Concentration (μM)
Coumarin	CYP2A6	25
Quinidine	CYP2D6	10
Quercetin	CYP2C8	20
α-Naphthoflavone	CYP1A2	10
Sulfaphenazole	CYP2C9	20
Ketoconazole	CYP3A4	1
Thioridazine hydrochloride	CYP2C19	25

**Table 3:** The nonspecific adsorption coefficient of ultrafiltration tube at TPB15.

Indicated Concentration (μg/mL)	C_pre_ (μg/mL)	C_post_ (μg/mL)	NSB (%)
4	4.01 ± 0.03	3.94 ± 0.05	1.75 ± 0.16
8	7.97 ± 0.09	7.75 ± 0.02	2.76 ± 0.27
16	16.09 ± 0.11	15.66 ± 0.13	2.67 ± 0.34

**Table 4:** Plasma protein binding rate of TPB15 in different species.

Indicated Concentration (μg/mL)	Plasma Protein Binding (%)				
	Human	SD Rat	KM Mice	Cynomolgus Monkey	Beagle Dog
4	81.51 ± 0.44	81.70 ± 0.10	81.80 ± 0.10	81.48 ± 2.00	81.48 ± 2.22
8	81.67 ± 0.15	80.87 ± 1.08	81.21 ± 0.34	81.24 ± 0.39	81.03 ± 0.51
16	82.15 ± 0.21	82.40 ± 0.14	82.40 ± 0.42	82.20 ± 0.85	82.28 ± 1.86

**Table 5:** Metabolic stability data of TPB15.

Species *	Regression Equation	R^2^	T_1/2_ (min)	CL_int_ (mL/min/mg)
RLM	Y = −0.0028 * X + 4.560	0.9218	305.15	0.0344
MouLM	Y = −0.0022 * X + 4.569	0.9346	241.55	0.0273
MLM	Y = −0.0078 * X + 4.526	0.9666	88.34	0.0941
DLM	Y = −0.0011 * X + 4.591	0.9377	630.00	0.0132
HLM	Y = −0.0067 * X + 4.529	0.9307	103.25	0.0805

* (RLM: Sprague-Dawley rat microsomes, MouLM: KM mice microsomes, DLM: beagle dog microsome, MLM: crab-eating monkey microsomes, HLM: human microsomes).

**Table 6:** Effect of each inhibitor on TPB15 in microsomal metabolism.

Inhibitor	CYP Isoform	Remaining Proportion of Substrate TPB15 (μg/mL)	RSD (%)	Inhibitor Rate (%)
Coumarin	CYP2A6	1.66 ± 0.12	7.32	6.60
Quinidine	CYP2D6	1.63 ± 0.04	2.88	4.82
α-Naphthoflavone	CYP1A2	1.64 ± 0.05	3.55	5.58
Quercetin	CYP2C8	1.68 ± 0.08	4.79	8.63
Sulfaphenazole	CYP2C9	2.64 ± 0.10	3.93	81.22
Ketoconazole	CYP3A4	2.23 ± 0.15	6.83	50.25
Thioridazine hydrochloride	CYP2C19	1.61 ± 0.10	6.11	2.79
Positive control group	—	1.57 ± 0.09	1.32	0.00
Negative control group	—	2.88 ± 0.04	1.44	—

## Data Availability

Data will be made available on request.

## Supplementary Materials

The following supporting information can be downloaded at: https://www.mdpi.com/article/10.3390/pharmaceutics17040423/s1, Table S1. Plasma metabolism of TPB15 in different species; Table S2. Metabolic data of testosterone in various liver microsomes.

## Abbreviations

The following abbreviations are used in this manuscript:

TNBC	Triple-negative breast cancer
Hh	Hedgehog
CYP450	Cytochrome P450
Gli	Glioma-associated oncogene
GPCRs	G-protein coupled receptors
*IC*_50_	Half-maximal inhibitory concentration
LD_50_	Lethal dose for 50% of the population
