# Supplementary material for: Plasma Protein Binding, Biostability, Metabolite Profiling, and CYP450 Phenotype of TPB15 Across Different Species: A Novel Smoothened Inhibitor for TNBC Therapy
Source: Pharmaceutics. 2025 Mar 26;17(4):423. doi: 10.3390/pharmaceutics17040423 (PMC12030497; doi:10.3390/pharmaceutics17040423)
Supplement: Supplementary file 1 [file pharmaceutics-17-00423-s001.zip › pharmaceutics-3497392-supplementary.pdf]

Table S1. Plasma metabolism of TPB15 in different species.

Incubation time (min)	Remaining proportion of substrate TPB15（%）				
	Human	SD rat	KM mice	Cynomolgus monkey	Beagle dog
0	100.0 ± 1.0	100.0 ± 0.6	100.0 ± 0.7	100.0 ± 1.7	100.0 ± 1.1
30	97.3 ± 1.5	99.7 ± 1.5	98.6 ± 0.9	98.2 ± 0.6	99.3 ± 0.7
60	97.7 ± 0.6	97.6 ± 0.6	98.0 ±1.5	96.3 ± 1.2	98.9 ± 0.4
90	98.0 ± 1.0	99.1 ± 0.3	97.1 ± 0.7	99.3 ± 0.9	97.1 ± 1.4
120	97.2 ± 1.1	98.0 ± 0.6	98.0 ± 1.2	98.3 ± 1.5	98.0 ± 0.8

Table S2. Metabolic data of testosterone in various liver microsomes.

Time（min)	Serial Number	Concentration（ng/mL）				
		RLM	MouLM	MLM	DLM	HLM
0	1	331	400	293	300	273
	2	331	314	385	364	379
	3	303	356	301	375	308
	Mean	321.7	356.7	326.4	346.3	320.0
	SD	16.2	43.0	50.9	40.5	54.0
90	1	33.6	2.7	29.2	19	19.2
	2	24.2	6.1	39.7	9.1	22.9
	3	9.8	24.4	38.2	24.4	34.7
	Mean	22.6	10.2	35.7	17.5	25.6
	SD	12.0	12.7	5.7	7.8	8.1
